# Gradient Descent Optimization in Gene Regulatory Pathways

**DOI:** 10.1371/journal.pone.0012475

**Published:** 2010-09-03

**Authors:** Mouli Das, Subhasis Mukhopadhyay, Rajat K. De

**Affiliations:** 1 Machine Intelligence Unit, Indian Statistical Institute, Kolkata, India; 2 Department of Bio-Physics, Molecular Biology and Bioinformatics, University of Calcutta, Kolkata, India; Albert Einstein College of Medicine, United States of America

## Abstract

**Background:**

Gene Regulatory Networks (GRNs) have become a major focus of interest in recent years. Elucidating the architecture and dynamics of large scale gene regulatory networks is an important goal in systems biology. The knowledge of the gene regulatory networks further gives insights about gene regulatory pathways. This information leads to many potential applications in medicine and molecular biology, examples of which are identification of metabolic pathways, complex genetic diseases, drug discovery and toxicology analysis. High-throughput technologies allow studying various aspects of gene regulatory networks on a genome-wide scale and we will discuss recent advances as well as limitations and future challenges for gene network modeling. Novel approaches are needed to both infer the causal genes and generate hypothesis on the underlying regulatory mechanisms.

**Methodology:**

In the present article, we introduce a new method for identifying a set of optimal gene regulatory pathways by using structural equations as a tool for modeling gene regulatory networks. The method, first of all, generates data on reaction flows in a pathway. A set of constraints is formulated incorporating weighting coefficients. Finally the gene regulatory pathways are obtained through optimization of an objective function with respect to these weighting coefficients. The effectiveness of the present method is successfully tested on ten gene regulatory networks existing in the literature. A comparative study with the existing extreme pathway analysis also forms a part of this investigation. The results compare favorably with earlier experimental results. The validated pathways point to a combination of previously documented and novel findings.

**Conclusions:**

We show that our method can correctly identify the causal genes and effectively output experimentally verified pathways. The present method has been successful in deriving the optimal regulatory pathways for all the regulatory networks considered. The biological significance and applicability of the optimal pathways has also been discussed. Finally the usefulness of the present method on genetic engineering is depicted with an example.

## Introduction

Gene regulatory networks perform fundamental information processing and control mechanisms in the cell. Regulatory genes code for proteins that activate or inhibit the expression of other genes, thereby forming a complex web of interactions. Such networks perhaps form the most important organizational level in the cell, where signals from the cell state and the outside environment are integrated in terms of activation and inhibition of genes. Genetic network analysis [1] is expected to help experimental biology in many ways. Practical applications may have a strong impact on biotech and pharmaceutical industries, and in genetic engineering, potentially setting the stage for rational redesign of living systems and predictive model-based drug design [2].

Owing to the high connectivity of the different regulatory interactions within the gene regulatory network, there has been considerable interest in exploiting tools from functional genomics for mapping of global regulatory structures or using high throughput experimental techniques for determining how regulatory flows through different branches of the gene regulatory network are controlled. Regulatory flows through a given interaction can be controlled by transcription, translation or posttranslational modifications, i.e. modification of the active enzyme concentration. The activity of genes in genomes of higher eukaryotic organisms is regulated mainly by the means of huge class of regulatory proteins (transcription factors, TF), through specific regulatory sequences - TF binding sites that are located usually in a proximity of the genes.

Pathway analysis is becoming increasingly important for assessing inherent network properties of biochemical reaction networks [3], [4]. Of the two most promising concepts for pathway analysis, one relies on elementary flux modes [5] and the other on extreme pathways. Flux balance analysis (FBA) [6] is based on the fundamental law of mass conservation and the application of optimization principles to determine the optimal distribution of resources within a network. Due to the presence of the inequality constraints on various fluxes, linear algebra can no longer handle such a mathematical system of equalities/inequalities, forcing the use of convex analysis [7], [8] to study the properties of the solution space. The mathematical foundations and unique features of these pathways enable one to evaluate pathway/network properties such as product yield, network robustness. Thus elementary modes and extreme pathways play a growing role in the analysis of complex biochemical reaction networks [9].

Flux balance analysis (FBA) has been useful for large scale analysis of metabolic networks, and methods have been developed to extend this approach for transcriptional regulation [10], [11]. Here we develop a method incorporating the principle of regularization for identification of an optimal pathway in gene regulatory networks starting from a given gene to a target gene. The method, first of all, generates the possible flow vectors in the pathway. We consider only those flow vectors which, by taking convex combination of the basis vectors spanning the null space of the given node-edge incidence matrix, satisfy the quasi-steady state condition along with other inequality constraints. Then a set of weighting coefficients representing concentration of various transcription factors is incorporated. A set of constraints involving these weighting coefficients is formulated. An objective function, in terms of these weighting coefficients, is formed, and then minimized under regularization method. The weighting coefficients corresponding to a minimum value of the objective function represent an optimal pathway. These optimal pathways determine the gene regulatory routes leading from the transcription of a given gene to the transcription of another gene, and represent the structural and functional properties of the network as a whole. The methodology can be viewed as flow of some information (or some approximation thereof) in a regulatory network, and an optimal path means the pathway where disruption has the largest effect. The effectiveness of the present method is demonstrated on ten gene regulatory networks. The results are compared with those obtained from the existing extreme pathway analysis [12], [13]. Results have been validated appropriately from biological point of view.

The exploration of optimal regulatory pathways helps in understanding the extent of regulatory relationships among the genes. Through this study, it is possible to compare optimal regulatory pathways over various stages of development, and a variety of other cellular phenotypes over diseases [14], [15]. Inferring the genes on the optimal regulatory path is challenging and very important in disease studies [16]. These regulatory pathways have been widely found in multiple biological processes and are considered to be one of the most fundamental gene expression regulatory mechanisms in biological systems [17]. This method might be successful in identifying important genes that are responsible for ceratin diseases [18], [19]. Genes on the optimal regulatory pathway have immediate and widespread interest as markers for diseases [15]. Precise knowledge of optimal gene regulatory pathways can provide an understanding of the time-dependent enhancement and suppression of gene activity and drug effectiveness [20]–[22].

## Results

**Figure 1:**
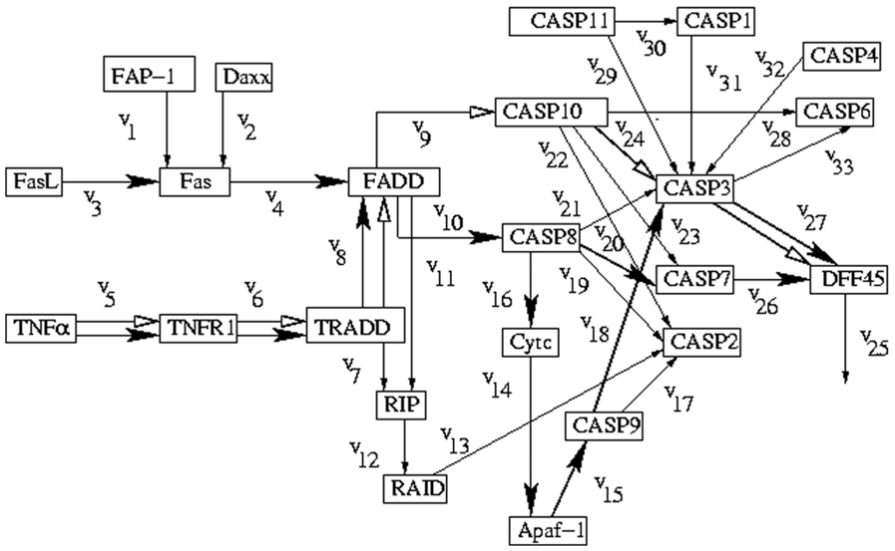
Path diagram for apoptotic genetic network. Two optimal regulatory pathways obtained by the present method are shown by bold black arrows, and one extreme regulatory pathway obtained by the extreme pathway analysis is shown by white arrows.

**Figure 2:**
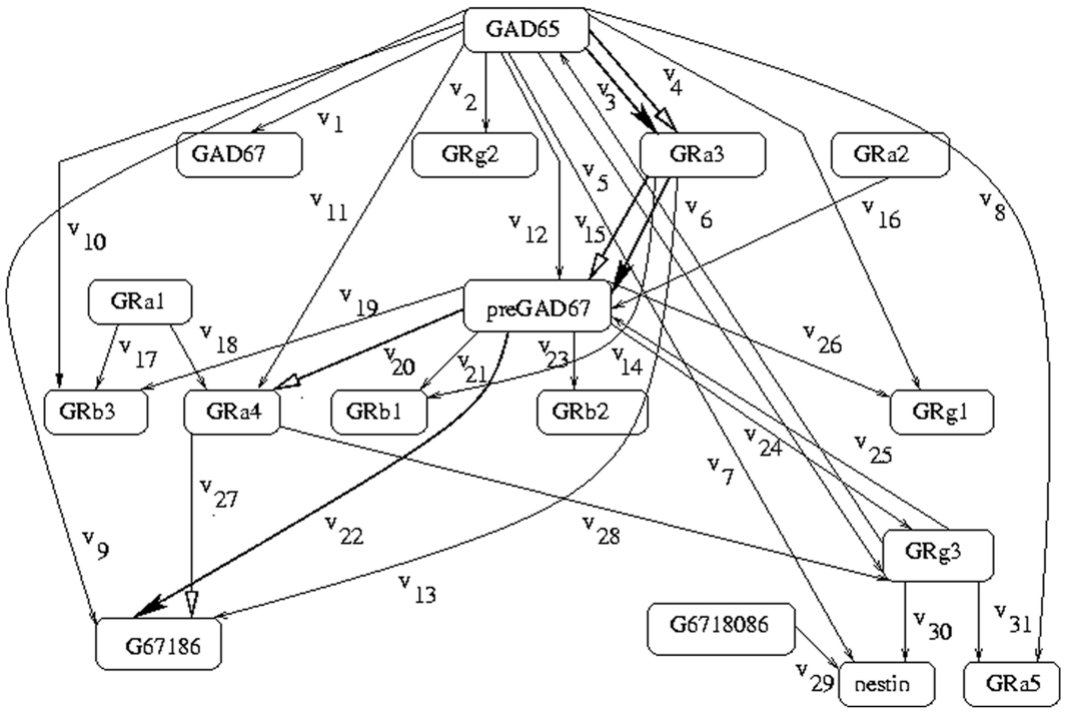
Path diagram for the subnetwork indicating the main interactions between GAD and GABA-receptors during the development of rat cervical spinal cord. The optimal regulatory pathway is shown by bold black arrows and the extreme regulatory pathway is shown by white arrows.

**Figure 3:**
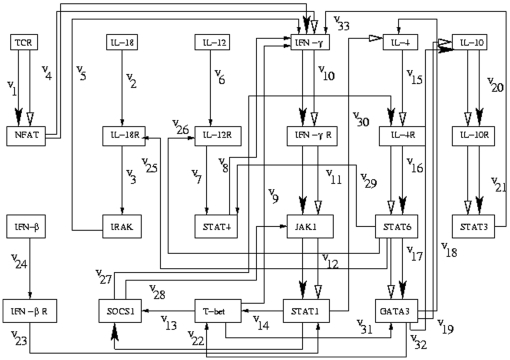
Path diagram for the Th regulatory network that controls the differentiation of Th cells in human. The optimal regulatory pathway is shown by bold black arrows and the extreme regulatory pathway is shown by white arrows.

**Figure 4:**
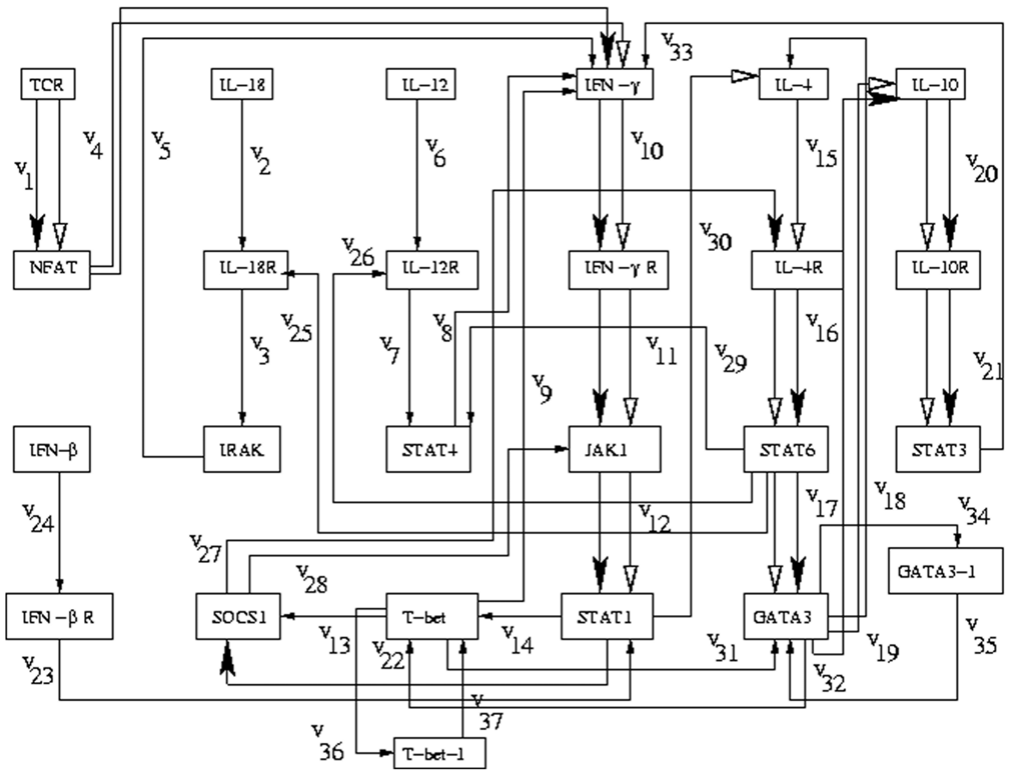
Path diagram for the Th regulatory network that controls the differentiation of Th cells in mouse with feedback. The optimal regulatory pathway is shown by bold black arrows and the extreme regulatory pathway is shown by white arrows.

Here we demonstrate the effectiveness of the present method using various gene regulatory networks. For this purpose, we consider ten gene regulatory networks as shown in Fig. 1 [13], Fig. 2 [23], Fig. 3 [24], Fig. 4 [25] and Fig. S1 [13], Fig. S2 [26], Figs. S3, S4, S5 [27] and Fig. S6 [28]. The results have been compared with that of the existing extreme pathway analysis [12], [13]. Biological validation of the results is also included.

It may be mentioned here that the present method involves a parameter 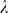, called Lagrange's multiplier or regularizing parameter. We vary the value of 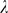 from 0.1 to 1.0. Initially, we should always give the stress on the maximal expression of the target gene which is our ultimate objective. That is, as it is seen from equation (4), initially 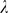 should be kept small. As we go from 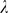 = 0.1 to 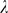 = 1.0, it implies that we are increasing the stress on the constraint, and finally both the amount of yield (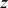) and the constraint are treated equally. For each value of 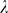, we minimize the objective function of equation (4) where 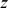 is given by equation (1) to obtain a proper set of values for 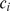
's for which 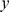 attains a minimum value. We consider that set of 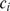-values corresponding to 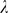 as the final solution, for which 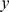 becomes minimum. Indeed it can be seen that 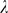 can be legitimately be called the regularizing parameter.

The regulatory network can be formulated by representing it as reactions in the stoichiometric matrix and then the integrated network can be analyzed by using extreme pathway analysis. The main difference to this work is that modeling based on the stoichiometric matrix requires a flux through the regulatory network. This approach is valuable for identifying underlying regulatory pathways in a regulatory network. Models such as regulatory FBA attempt to explicitly model regulation by switching fluxes on and off, based on the experimental data of enzyme expression in various growth conditions.

A complete picture of cellular regulation must take into account metabolic reactions and their interplay with the regulatory layer. Regulated flux balance analysis (rFBA) is a modeling approach that aims to integrate regulation and metabolism. A major problem in using ordinary differential equations (ODEs) for describing biochemical reactions is the scarcity of experimental data on rate constants. rFBA addresses this problem by assuming that the network is in a steady state and therefore that the total concentration of each substance does not change under this assumption, a system of ODEs is transformed into a system of linear equations, and its rates can be obtained by solving a linear programming problem that optimizes a certain objective function. Such optimization problems can be solved efficiently. Further constraints are added to narrow the solution space. For example, the rate constants are restricted according to the catalytic capacities of transcription factors. The method has been successfully used to model large regulatory networks covering the near complete regulation of several species.

A major difficulty of modeling regulatory networks is the context-specific nature of gene regulation. The total space of possible transcriptional regulatory interactions for an organism is the number of transcription factors multiplied by the number of genes multiplied by the number of environmental contexts in which the cell might find itself.

Here we have explored a framework for modeling transcriptional regulatory networks in which experimental design and validation are central features. This framework is based on computational analysis suggesting a high-throughput strategy for mapping gene-regulatory pathways.

### Apoptotic Genetic network

The genetic network in Fig. 1 represents a part of apoptosis regulation [13]. Apoptosis is one of the main types of programmed cell death, which involves a series of biochemical events leading to specific cell morphology, characteristics and ultimately death of cells. A family of proteins known as caspases is activated in the early stages of apoptosis [29]. Induction of apoptosis via death receptors typically results in the activation of an initiator caspase such as CASP 8 or CASP 10. These caspases can then activate other caspases in a cascade. This cascade eventually leads to the activation of the effector caspases, such as CASP 3 and CASP 6. These caspases are responsible for the cleavage of the key cellular proteins, such as cytoskeletal proteins, that leads to the typical morphological changes observed in cells undergoing apoptosis. There are 23 genes, 33 internal flows and no external flows present in Fig. 1.

The starting genes are 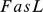 and 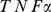, and the target gene is 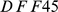 (Fig. 1). Here 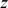 is defined as 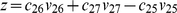. Following the method described in Section Method, we have obtained the 2 optimal regulatory pathways as 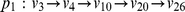, 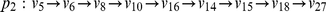 as shown by bold black arrows. These are the two major experimentally confirmed pathways (extrinsic and intrinsic apoptosis pathways) 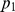 and 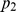 [30] through which apoptosis can be triggered in a cell. The extreme regulatory pathway obtained by the extreme pathway analysis is different from that obtained by the proposed method and is as follows 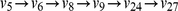 as shown by white arrows.

Table S1 shows a few pathways from the starting gene to the target gene along with 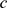-values and the average amount (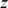) of the protein synthesized by the target gene 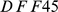. Since, we have generated a set of flow vectors, we have considered average of these vectors to compute the average amount of the protein synthesized (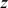). For example, the pathways 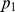 and 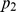 corresponding to serial number 4 and 5 in Table S1 yield the highest average 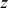, and hence these are the optimal regulatory pathways. It can be inferred from Table S1 that the corresponding 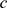-values for the pathways 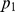 and 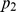 are larger compared to the other 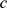-values of other pathways. Thus it can be inferred from the 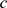-values and the 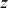-values that the present method is able to correctly identify the optimal gene regulatory pathways.

We have varied the upper bound of the flow values to show the variation of transcription factors (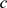-value) and the amount (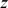) of the protein synthesized by the target gene. The results are provided in Table S2 for some high and low upper bounds. It is clear from Table S2 that 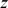-value, as expected, decreases with the decrease in upper bound. In all the cases, we have found the same optimal path although absolute 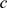-values differ. This shows the consistency of the present method in determining optimal gene regulatory paths.

### Genetic network for the development of rat cervical spinal cord

Fig. 2 is a genetic network connecting 65 mRNA species during the development of rat cervical spinal cord. The figure represents the interaction of GAD (glutamic acid decarboxylase) and GABA-R (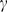-amino butyric acid receptors). In a rat, two forms of GAD exist, GAD65 and GAD67, as shown in Fig. 2. GABA, synthesized from glutamate by GAD, is a well-known fast-acting synaptic transmitter in the mature CNS [23]. However, it is also thought to play an important role in CNS differentiation during early CNS development.

In Fig. 2, the starting gene is 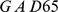 and the target gene is 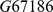. There are 17 genes and 31 interactions in the network. The expression for 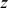 is given by 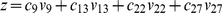. Here an optimal pathway has been found to be 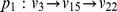 as shown by bold black arrows. The extreme regulatory pathway obtained by the extreme pathway analysis is different from that obtained by the present method and is as follows 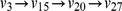 as shown by white arrows.

### Th regulatory network of human

Fig. 3 represents the Th regulatory network that controls the differentiation of T-helper (Th) cells. Here the starting gene is 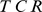 and the target gene is 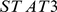. The immune system of our body contains diverse cell populations such as antigen presenting cells, natural killer cells, B and T lymphocytes. T lymphocytes are classified as either T helper cells (Th) or T cytotoxic cells (Tc). T helper cells take part in cell and antibody-mediated immune responses by secreting various cytokines, and they are further sub-divided into precursor Th0 cells, and effector Th1 and Th2 cells, depending on the array of cytokines that they secrete [31]. The network that controls the differentiation from Th0 towards the Th1 or Th2 phenotypes is a complex network [25]. Here we have used an updated version of the Th network in human where there is no feedback loop. There are 33 reactions and 23 genes in the network. Here we have 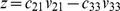. An optimal pathway obtained by the present method is 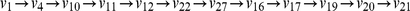 as shown by bold black arrows. The extreme regulatory pathway obtained by the extreme pathway analysis is different from that obtained by the present method and is as follows 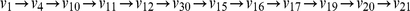 as shown by white arrows.

### Regulatory networks with feedback: Th regulatory network of mouse

The genes GATA3 and T-bet in the Th regulatory network of mouse (Fig. 4) as considered by Mendoza in [25] include a self-activation loop. In order to incorporate these feedback loops in our methodology, we have considered two hypothetical nodes GATA3-1 and T-bet-1 analogous to the nodes corresponding to the genes GATA3 and T-bet. Thus the order of the node-edge incidence matrix becomes 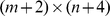, where 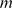 is the number of genes and 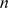 is the number of regulatory interactions. The optimal regulatory pathway obtained by our method after incorporating these two hypothetical genes remains the same as in the case of Fig. 3. This is due to the fact that 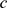-values corresponding to the edges connecting actual and hypothetical nodes are found to be small compared to that of the other edges. The extreme regulatory pathway also remains the same as before.

### Biological relevance and validation

Here we provide relevance and validation of the results from biological point of view. For this purpose, we have searched the literature, and validation of the results is made based on the results obtained by earlier investigations.

### Apoptotic Genetic network

Apoptosis is a complex process that proceeds through at least two main pathways (extrinsic and intrinsic), each of which can be regulated at multiple levels. The extrinsic pathway consists of cell surface receptors, their inhibitory counterparts and their associated cytoplasmic proteins. The intrinsic pathway centers on the mitochondria, which contain key apoptogenic factors such as cytochrome c, AIF, SMAC/DIABLO, Htra2/Omi and endoG.

In the case of apoptotic genetic network, the biological significance of the two major experimentally confirmed pathways (extrinsic and intrinsic apoptosis pathways) 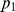 and 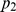 [30] as obtained by the present method in Fig. 1 is described here. The pathway 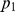 has FasL as the initial gene and DFF45 as the target gene. There are three paths emerging from the intermediate gene FADD. The path involving the flows 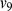 to 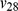 is not followed as it does not lead to the target gene DFF45. There are three paths emerging from the intermediate gene CASP10. The path involving 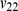 is not followed as it does not yield the target gene. Though the other two paths involving 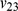 and 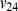 yields the target gene but they are not followed. The other path from FADD through 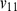 is not followed as it leads to the formation of the gene CASP2 which is not the desired target gene. The occurrence of the gene FADD has been observed in [32]–[34]. The optimal regulatory path leads from FADD to CASP8 whose occurrence has been demonstrated in [35]. There are four paths emerging from the intermediate gene CASP8. The path through 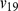 is not followed as it does not yield the target gene. We reach the target gene through the flow 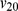. The existance of the path through 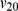 and 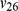 to yield the target gene is established in [36], [37] in contrary to the other two paths through 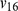 and 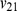. Moreover, the path through 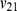 yielding CASP3 as the intermediate gene cannot be followed and has been explained in [38]. The extrinsic apoptotic pathway 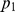 as derived by the regularization method has been observed in [39]–[42].

The pathway 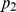 has TNF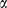 as the initial gene and DFF45 as the target gene. After reaching the intermediate gene TRADD the path divides into two branches. The occurrence of the gene TRADD in the apoptotic path has been observed in [43]. The path through 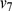 is not followed as it ultimately terminates to the gene CASP2 which is not the desired target gene DFF45. The path through 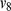 is followed. From the intermediate gene FADD, three paths emerge of which the path through 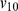 is followed till we reach the intermediate gene CASP8. It has already been explained in the previous paragraph that the other two paths through 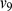 and 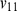 are not followed. There are four paths emerging from CASP8. The path through 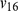 yielding Cytc, Apaf-1 and CASP9 is followed. From CASP9 the path through 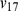 is not followed as it terminates to the gene CASP2, which is not the desired target gene. So the path through 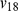 yielding CASP3 as the intermediate gene is followed. From CASP3, the path through 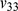 is not followed as it terminates to the gene CASP6, which is not the target gene. So the path through 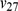 yielding DFF45 as the target gene is followed. Of the remaining three paths from CASP8, the path through 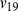 is not followed as it terminates to the gene CASP2 that is not the desired target. The path through 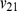 yielding CASP3 as the intermediate gene cannot occur biologically and has been explained in the previous paragraph. The path through 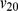 yielding CASP7 as the intermediate gene is a subpath of the extrinsic pathway. The intrinsic apoptotic pathway 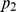 as derived by the present method has been observed in [40], [41].

Starting from the gene TNF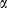, both the extreme pathway analysis and the present method follow the same path till they arrive at the intermediate gene FADD. The existance of the optimal pathway through the genes TNF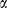, TNFR1, TRADD and FADD has been observed in [44]–[46]. From FADD, the path obtained by our present method coincides with the intrinsic and the extrinsic pathway and not the path obtained by extreme pathway analysis. The intrinsic path that leads from FADD to the target gene DFF45 through the intermediate path as obtained by the present method can be found in [47]–[49].

The acquired biological knowledge of the apoptosis regulatory network can be translated into mathematical models, in particular focusing on the regulatory events. Two distinct modeling approaches i) Modeling by deterministic ODEs and ii) stochastic CA-based (cellular automation) models that determines regulatory pathways from experiments exist in the literature. The pathways obtained by our method coincide with those pathways determined by both ODE and CA-based models for the apoptosis regulatory network [50].

### Genetic network for the development of rat cervical spinal cord

In Fig. 2, there are 11 paths emerging from the starting gene GAD65 of the genetic network for the development of rat cervical spinal cord. The paths through 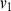, 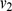 and 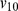 are not followed as they terminate to the intermediate genes GAD67, GRg2 and GRb3 respectively, which are not the desired targets. The paths through 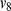 and 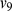 do not lead to the optimal path. The path following 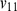 terminates to the intermediate gene GRa4. From GRa4, we can reach the target gene G67186 through 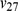 but this path is not followed. The other path through 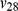 from GRa4 is not followed as it terminates to another intermediate gene GRg3 that is not the required target. Another path through 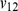 reaches the intermediate gene preGAD67. From preGAD67, the paths leading through 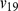, 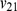, 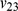, 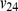 and 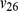 are not followed as they terminate to some intermediary genes and are not the target gene. The path through 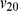 from preGAD67 ultimately leads to the target gene by the flow 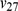 but is not followed. The path through 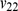 does not lead to the optimal path. The paths through 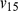, 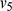 and 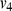 do not reach the desired target and hence are not the optimal paths. The path leading through 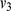 is followed till we reach the intermediate gene GRa3. Of the 3 paths emerging from GRa3, the paths through 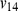 ends up at an intermediate gene GRb1 and the other path through 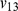 is not the optimal path. So the only remaining path from GRa3 through 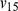 is followed which ultimately leads to the target gene G67186 by the flow 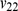, and this sequence of steps forms the desired optimal regulatory pathway. The importance of the starting gene GAD65 and the intermediate gene GRa3 in the optimal regulatory pathway has been observed in [51], [52]. The pathway obtained by the present method follows [53], in contrary to the path obtained by the extreme pathway analysis.

### Th regulatory network

The Th regulatory network in Fig. 3 has TCR as the starting gene and STAT3 as the target gene. The biological significance of the path that we have derived by our algorithm is described here. The path follows from TCR through NFAT, IFN-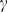, IFN-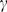
R, JAK1 till we reach the intermediate gene STAT1. The path gets divided into three branches at the intermediate gene STAT1. The path through 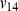 is not followed as the path from another intermediate gene SOCS1 through 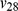 follows a self loop. So the path through 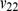 is followed through SOCS1, IL-4R, STAT6, which is the same as obtained from our method till we arrive at another intermediate gene GATA3. There are three paths emerging from GATA3. The paths through 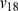 and 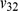 are not followed as they end up in a loop structure. So the path through 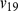 is followed to reach the target gene which is the same as obtained from our present method and is found in [54], [55].

Selective activation of T helper (Th) cell subsets plays an important role in the pathogenesis of human allergy and inflammatory diseases. Dissecting pathways and regulatory networks leading to the development of Th1 or Th2 cells will be crucial to understand the pathogenesis of allergy and inflammatory diseases. Improved understanding may lead to better strategies for developing diagnostics and effective therapies for these diseases. The recent results have led to novel hypotheses on the transcription factors involved in human Th cell differentiation. Effort has been given at elucidating the function of the novel genes and pathways identified from literature with primary human CD4+ T cells. Detailed analysis of upstream T cell Receptor (TCR)/key cytokine receptor induced regualtory pathways includes repeated rounds of mathematical modelling and experimental verification. The signalling and transcriptional protein complexes are analyzed with mass spectrometry and cell imaging techniques to build a model of T cell activation and differentiation.

### Prostate genetic network, Multiple-myeloma (MM) tissue genetic network and SOS genetic network

The target gene CAV1 on the optimal regulatory path in Fig. S3 (in the Prostate genetic network in Text S1) was involved in breast cancer [56] and ovarian carcinoma [57]. It was reported that the gene DF on the optimal regulatory path in Fig. S5 (in the (MM) tissue genetic network in Text S1) was a novel serine protease [58] and was involved in myeloid cell differentiation [59]. The gene AX1 on the optimal regulatory path was a tyrosine kinase receptor and was recently found down regulated in mature bone marrow-derived dendritic cells [60].

The SOS pathway in Fig. S6 (in the SOS genetic network in Text S1), which regulates cell survival and repair after DNA damage, involves the lexA and recA genes [1]. There are 3 paths emerging from the starting gene lexA in Fig. S6. The paths leading through 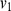 and 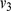 are not followed as they terminate to the intermediate genes umuDC and dinI which are not the required targets. The only remaining path through 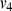 is followed till the intermediate gene ssb is reached. There is a single path from ssb leading to the target gene rpoD through 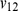, which is the desired optimal regulatory pathway. The importance of the starting gene lexA, the intermediate gene ssb and the target gene rpoD has been observed in [28], [61], [62].

### Discussions on the present method: Impact on genetic engineering

The computational prediction of all biologically relevant or novel alternative routes in regulatory networks has numerous applications in systems biology. The present method can be applied to maximize/minimize the amount of a target product by expressing/inhibiting optimal pathways, under the framework of genetic engineering. Here we describe briefly such a problem on the production of fermentative hydrogen and show how the present method may be applicable to this problem.

**Figure 5:**
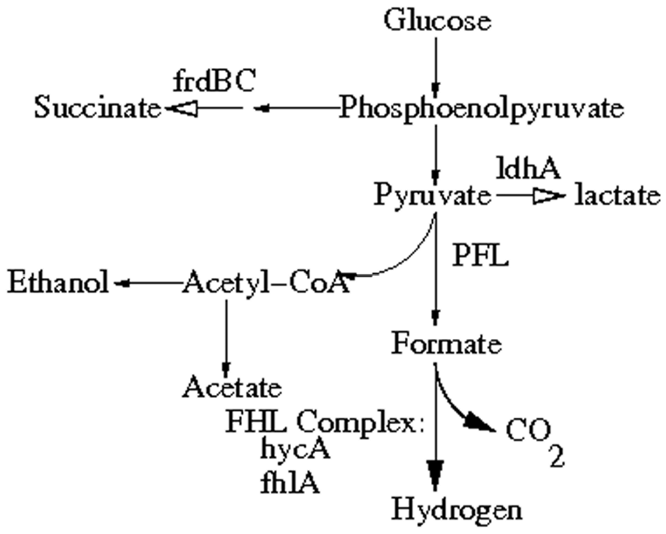
Fermentative hydrogen production pathway from glucose by *E. coli*. The bold white arrows are the pathways inactivated by disrupting ldhA and frdBC, and the bold black arrows are the pathways enhanced by disrupting hycA and over expressing fhlA.

Microorganisms produce hydrogen via two main pathways: photosynthesis and fermentation. Here we consider microbial production of hydrogen by fermentation (more advantageous than the photosynthetic hydrogen production) and provide an overview to enhance fermentative hydrogen production through genetic engineering. We have chosen to genetically engineer *E. coli* [63] for hydrogen production as this is the best-characterized bacterium (i.e. has well-established metabolic pathways) and it is one of the easiest strains to manipulate genetically. The fermentative route of hydrogen production in *E. coli* (Fig. 5) starts with the conversion of glucose to pyruvate, which is then converted to acetyl-CoA and formate, which is catalysed by pyruvate formate lyase (PFL). Biological hydrogen production from formate is catalyzed by the formate hydrogen lyase (FHL) complex. The FHL complex of *E. coli* has been the most extensively characterized at both the physiological and genetic levels.

Hydrogen is produced from glucose by fermentation with the simultaneous release of carbondioxide which is not hydrogen hogging, instead of water which is released during photosynthesis. As we proceed along the hydrogen producing pathway the intermediate steps leading to production of succinate, lactate, acetate and ethanol involve hydrogen. As our ultimate goal is to maximize the target product hydrogen we have to disrupt/block the hydrogen hogging pathways and enhance the pathways that produce hydrogen. It has been observed from our methodology that the values of the flux vectors along the path that yields hydrogen from glucose via pyruvate gradually increases in contrary to the values of the fluxes that gradually decreases along the intermediate hydrogen hogging pathways (Table 1). This ultimately leads to maximal production of hydrogen from glucose via pyruvate simultaneously blocking other intermediate steps that produce succinate, lactate, ethanol and acetate. Thus we can conclude that our proposed methodology has been successful in deriving the optimal path from glucose to generate maximum amount of hydrogen.

**Table 1:** Values of flux vectors for the system in Fig. 5.

Serial Number	Intermediate steps in the pathway	Flux vector 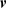
**1**	Glucose 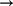 Phosphoenolpyruvate	19.57
**2**	Phosphoenolpyruvate 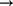 Pyruvate	26.73
**3**	Phosphoenolpyruvate 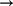 Succinate	8.75
**4**	Pyruvate 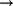 Lactate	7.34
**5**	Pyruvate 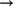 Acetyl-CoA	6.92
**6**	Acetyl-CoA 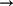 Ethanol	5.22
**7**	Acetyl-CoA 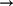 Acetate	5.06
**8**	Pyruvate 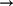 Formate	30.45
**9**	Formate 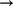 Hydrogen	35.29

The fermentative hydrogen metabolism in *E. coli* is determined by 50 genes distributed across 20 distinct genetic loci [64]. The modification of transcriptional regulators and enzymes are needed for the coordinated engineering of genes and operons that perform distinct biochemical functions related to the production of hydrogen. Here Fan *et. al.* have described a method for achieving increased molar yield of hydrogen by modifying certain genes involved in the pathway that produces hydrogen from glucose under anaerobic conditions and globally regulate the fermentative hydrogen production in *E. coli*.

There are two possible ways through which improved hydrogen yields from glucose can be achieved. The first involves directing glucose metabolism toward pyruvate formate lyase (PFL) by disrupting the succinate-producing and lactate-producing pathways. The second encompasses enhanced downstream pathways of PFL through overexpression of the formate hydrogen lyase (FHL) complex. Since the genes fhlA and hycA control the transcription of the FHL complex, it is theoretically possible to control the specific FHL activity and the specific hydrogen production rate by manipulating these genes or their genetic controls. The fermentative biohydrogen production from formate can be increased by overexpressing the FHL activator encoded by the fhlA and by inactivating the FHL repressor encoded by the hycA in *E. coli* K-12 strain W3110. The present method becomes useful if we can increase the transcription factor and hence increase the expression level of the gene fhlA and decrease the transcription factor for the gene hycA for the corresponding optimal regulatory path. Moreover, it has been experimentally observed in [65], [66] that the hydrogen production rate was 2.8-fold higher with both fhlA overexpressed and hycA inactivated in *E. coli* K-12 strain W3110.

Enhanced hydrogen yield from glucose can also be obtained by blocking the competing lactate (via deleting the gene ldhA) and succinate (via deleting the gene frdBC) production pathways. Our method becomes effective for this case if we can decrease ldhA and frdBC, and/or their transcription activators for ldhA and frdBC. Thus it can be concluded that blocking some pathways (decreasing the expression levels of the genes and/or their transcription activators in the path) through mutagenesis results in enhanced hydrogen production from glucose.

If the transcription factors affect the target gene(s) positively, then the expression level(s) of the target gene(s) increase and vice versa. Our method becomes useful if we can increase the transcription factor and hence increase the expression level of the gene(s) to make that pathway active. If we want to switch off any pathway we have to reduce the gene expression level and hence decrease the transcription factor for that corresponding path. However, the present method can be useful for this example to determine an optimal regulatory pathway through which the amount of hydrogen is maximum. We can apply the method to this problem for determining the optimal gene regulatory pathway and finally express this optimal path. Discovering novel optimal gene regulatory pathways through genetic engineering may also help to make biological hydrogen production more favorable, practical and commercially competitive.

## Discussion

Here we have developed a network based algorithm for exploring gene regulatory networks in which the underlying optimal regulatory pathways from a starting gene to a target gene can be determined in terms of concentration of various transcription factors regulating the genes in the network. In other words, the method determines an optimal set of transcription factors that need to be expressed to get an optimal gene regulatory pathway from starting gene(s) to target gene(s).

The effectiveness of the regularization method has been demonstrated on ten gene regulatory networks to infer optimal regulatory pathways which has practical applications in the field of genetic engineering. The significance of the optimal pathways has been biologically validated through extensive literature survey. Finally we have shown with an example how the method can be effectively used in the field of genetic engineering. As regulatory networks are reconstructed with a matrix formalism as presented herein, these analysis tools can be used to characterize fundamental features of such systems.

Information about gene regulatory pathways can be used to infer topological features and regulatory interactions of the network. However, it is known that regulatory pathways do not persist over all time. An important recent finding in which the above is seen to be true is following examination of regulatory networks during the yeast cell cycle, wherein topologies change depending on underlying (endogeneous or exogeneous) cell condition.

In order to describe the knowledge on regulatory pathways for simulation, a considerable amount of attention have been paid to Petri net for details. Petri net is a network consisting of place, transition, arc, and token. The conventional Petri net can be used to model only the discrete features in biological pathways, e.g. logical regulatory relationships between genes. But biological pathway modeling requires some continuous features with enzyme reactions represented with differential equations.

Cancer is a heterogeneous disease often requiring a complexity of alterations to drive a normal cell to a malignancy and ultimately to a metastatic state. In cancer research, microarray technology measures the gene expressions of cancer and normal tissues and identifies genes that are differentially expressed between cancerous and normal cells. The set of individual differentially expressed genes can only tell us which genes are altered by biological differences between different cell types and/or states. It cannot explain the reasons for the significant alterations in gene expression levels and the effects of such changes on other gene activities. In a biological system genes interact with each other forming various regulatory pathways in order to carry out a multitude of biological processes. To better understand the roles of these differentially expressed genes and their interactions in a complex biological system, a comprehensive pathway analysis is needed. Since the identification of regulatory pathways is significantly influenced by those differentially expressed genes from different datasets or different statistical methods, an integration of multiple cancer microarray datasets and identification of the most common pathways from these data would reveal key relationships between crucial genes in carcinogenesis.

## Methods

A gene regulatory network can be represented as a directed graph where the nodes represent genes and the directed edge represents the regulatory relationship between two connected genes. Let 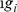 be the expression level of gene 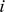 associated with node 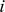 in the graph. There is a flow, associated with each directed edge 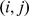 from node 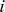 to node 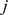, which indicates the flow of mRNA and thereby protein obtained from gene 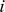 transported through the edge 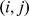. This protein now binds to gene 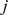 and regulates its expression level. It is to be mentioned here that we are using the flow of mRNA and proteins interchangeably. That is, we are considering only those fractions of mRNAs that are not degraded by any other factors, and form proteins through translation.

Here we present a method for identifying an optimal gene regulatory pathway from a starting gene to a target gene through which the expression level of the target gene becomes maximum. The genes on such an optimal pathway need to be expressed along with other transcription factors regulating them. Transcription factors bind to specific parts of DNA in the promoter region of a gene and, thus, affect the transcription of the gene. They can activate, enhance, or inhibit the transcription. Changes of abundances of transcription factors cause changes in the amount of transcripts of their target genes. This process is highly complex and interactions among transcription factors result in a more interwoven regulatory network.

The interactions among the genes describing their transcriptional regulation are considered as a matrix, called a node edge incidence matrix, 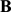. The order of the matrix is 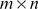 with 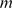 as the number of genes and 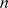 as the total number of regulatory interactions within a gene regulatory network. That is, the total number of edges is 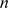. An element 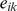 of matrix 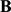 is −1 (+1) if 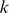-th edge (interaction) exits (enters) the node corresponding to gene 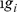. Otherwise, 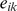 = 0. A system boundary is drawn around a gene regulatory network which consists of both internal and exchange flows. The internal flows are constrained to be positive and the exchange flows can be either positive if the flow enters the network, negative if the flow exits the network, or bidirectional. There are 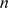 flows and 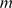 genes in the network. Let 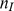 be the number of internal flows and 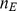 be that of exchange flows, and then 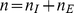. The 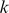-th internal flow is denoted by 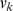 and the 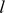-th exchange flow is denoted by 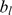. So there are 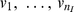 internal flows and 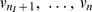 exchange flows where 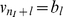.

**Figure 6:**
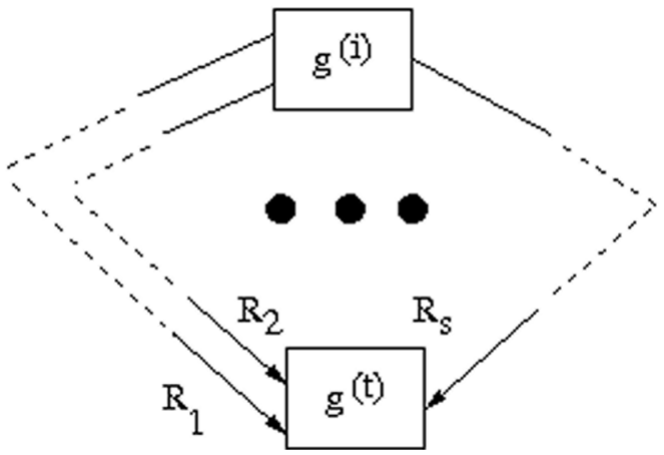
A hypothetical reaction network. The three dots indicates the continuation of the biochemical reactions from 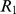 to 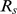 involving s different paths to reach the target gene. The reactions 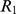, 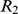 and 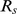, involving the target gene, are shown in the diagram.

The target gene 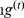 can be reached through various paths from the starting gene 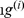 (Fig. 6). There are 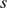 biochemical reactions/conversions 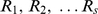 in the network involving the target gene 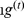.

We take the algebraic sum of the weighted flows of reactions 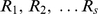 to reach the target gene 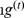, and it is given by
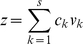
(1)which needs to be maximized for yielding maximum expression level of the target gene. The term 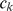 denotes the weighting factor, representing concentration of other transcription factors (not shown in the diagram) to get the corresponding flow 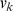. The proposed method involves three steps: (i) Generation of some flow vectors; (ii) Formulation of a new constraint; and (iii) Estimation of weighting coefficients 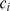.

There exists a well established methodology, called flux balance analysis, in the context of metabolic pathway analysis. In such analysis, a stoichiometric matrix is formed with the number of rows as the number of metabolites and the number of columns as the number of reactions (fluxes). The fluxes represent the rate of mass flow from one metabolite to the other through a reaction. We have extended this methodology to the analysis of gene regulatory networks. Here we consider node-edge incidence matrix 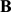, similar to stoichiometric matrix in case of metabolic pathways. The flux vectors are replaced by flow vectors where a component 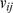 represents the flow of mRNA and thereby the protein produced from gene 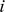 to gene 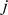. This protein becomes a transcription factor of gene 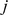 for its regulation. Thus, flow of mRNA and thereby proteins obtained from a gene and binding these proteins into another gene is considered as a chemical reaction as in the case of metabolic pathways.

### Generation of gene flow vectors

In this step, we generate some possible flow vectors for a gene regulatory network. The flow vectors satisfy approximately the quasi-steady state condition. That is, we generate those 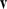 which satisfies
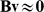
(2)where 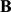 is the 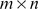 node-edge incidence matrix that describes the regulatory interactions among genes. 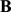 is computed from a given gene regulatory network. As 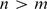, equation (2) is under determined. So we proceed in the following way:

a): Generate basis vectors 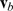 that span the null space of the node-edge incidence matrix 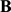. Let the number of such basis vectors be 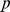. (This is done by standard functions available in MATLAB).

b): Generate 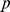 random numbers 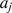, 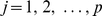. Then generate a vector 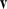 as a linear combination of the basis vectors using 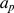 i.e., 
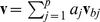
. We consider those 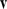 for which certain boundary conditions are satisfied for each of its components [12]. That is, 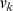 (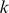
th component of 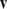) is an internal flow, 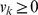. For 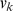 to be an exchange flow, 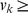
0 (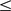
0), if the flow enters (exits) the network. If the exchange flow is bidirectional, 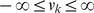.

### Incorporating feedback

**Figure 7:**
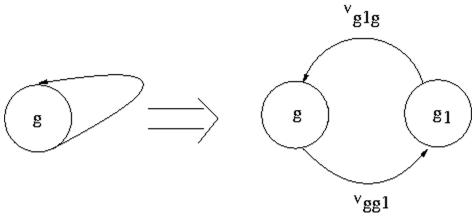
Incorporation of feedback loop. The feedback loop around the node corresponding to gene 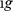 is replaced by considering a hypothetical node 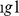, and edges (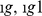) and (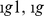).

A gene regulatory network often contains one or more feedback loop(s). In order to incorporate the effect of feedback possessed by a gene 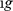, we consider a hypothetical node 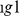, in addition to the node corresponding to gene 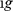 (Fig. 7), as it is not possible to put an entry corresponding to a feedback in the node-edge incidence matrix 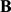. Then flows are made from 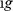 to 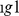 and 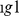 to 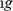. Thus the number of rows of 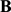 is increased by 1, and the number of columns of 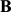 and the number of components of the flow vector are increased by 2 due to a single feedback. Now both 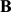 and the flow vectors 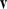 are generated by the above steps.

### Formulation of a new constraint

All the transcription factors that are not shown in a system may not be expressed at the required level so that the corresponding target genes may not be expressed/inhibited fully. This imposes further restrictions on the system and leads to variation in the concentration of other transcription factors. Thus we define a new constraint as
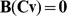
(3)where 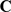 is an 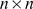 diagonal matrix, whose diagonal elements are the components of the vector 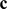. That is, if 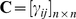, then 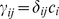, where 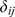 is the Kronecker delta. Thus the problem of determining an optimal regulatory pathway from a starting gene to a target gene boils down to an optimization problem, where 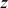 has to be maximized with respect to 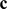, such that the aforesaid inequality constraints and the new constraint are satisfied.

### Estimation of weighting coefficients 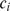


Combining equations (1) and (3), we can reformulate the objective function as
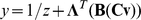
(4)that needs to be minimized with respect to the weighting factors 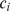 for all 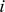. The term 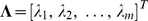 is called Lagrange's multiplier or regularizing parameter. For the sake of simplicity, we have considered here 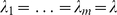 (say). Initially, a set of random values in 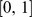 corresponding to 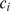
's are generated. Then 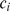
's are modified iteratively using gradient descent technique, where the amount of modification for 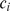 in each iteration is defined as
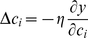
(5)The term 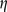 is a small positive quantity indicating the rate of modification. Thus the modified value of 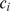 is 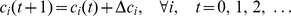 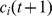 is the value of 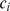 at iteration 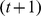, which is computed based on the 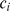-value at the iteration 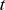.

Regularization parameter 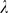 is chosen empirically from 0.1 to 1.0 in steps of 0.1. Using the above mentioned method, for each value of 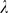, we finally get 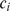-values for which 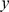 attains a minimum value. We choose a specific 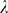 for which the 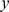-value is the minimum over all the minima attained for different values of 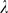. The concentration vector 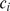 attains values between 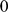 to 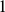 as mentioned previously corresponding to some values of 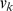 and is negligible for other values of 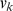. We take into account the values of 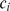
's that are close to 1, corresponding to the minimum value of 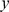. This enables us to identify the optimal regulatory pathway yielding the maximal expression of the target gene 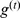 starting from the initial gene 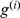.

## Supporting Information

Text S1 Supplementary information.
(0.08 MB PDF)

Table S1 Some possible pathways with their c-values and z-values for the system in Fig. 1.
(0.01 MB PDF)

Table S2 Variation of c-values and z-values with the upper bound on regulatory flows for the optimal path p1: v3→v4→v10→v20→v26 of the system in Fig. 1.
(0.01 MB PDF)

Figure S1 Path diagram for a genetic network reconstructed from yeast cell cycle data. The 3 optimal regulatory pathways are shown by bold black arrows.
(0.24 MB TIF)

Figure S2 Path diagram of three complex regulatory circuits of the extended transcriptional regulatory network of *E. coli*. The optimal regulatory pathways are shown by bold black arrows and the extreme regulatory pathway is shown by white arrows for part C.
(0.52 MB TIF)

Figure S3 Path diagram for prostate genetic network. The optimal regulatory pathway is shown by bold black arrows.
(0.27 MB TIF)

Figure S4 Path diagram for yeast cell cycle genetic network. The optimal regulatory pathway is shown by bold black arrows.
(0.23 MB TIF)

Figure S5 Path diagram for differentially regulated genetic network for the MM data set. The optimal regulatory pathway is shown by bold black arrows.
(0.24 MB TIF)

Figure S6 Path diagram of the interactions of the SOS network. The optimal regulatory pathway is shown by bold black arrows.
(0.19 MB TIF)
