# Supplementary material for: Gradient Descent Optimization in Gene Regulatory Pathways
Source: PLoS One. 2010 Sep 3;5(9):e12475. doi: 10.1371/journal.pone.0012475 (PMC2933224; doi:10.1371/journal.pone.0012475)
Supplement: Text S1 — Supplementary information. (0.08 MB PDF) [file pone.0012475.s001.pdf]

Supplementary Information

Linear Structural equation model

Gene regulatory networks can be described by ordinary differential equations (ODEs). The ODE approach provides detailed information about the network's dynamics, but requires high quality data on kinetic parameters and it is therefore currently applicable to only a few systems. Here we give some recent examples for modeling the dynamics of regulatory networks using ODEs. Here we are extending the concept of network-based metabolic pathways to gene regulatory networks and developing mathematical procedures for network-based regulatory pathway analysis. There are efforts taken to develop experimental and computational methods for defining the organization and function of the gene regulatory networks.

Gene regulatory networks are not static entities but dynamic structures that are expected to undergo significant topological changes in response to variations in cellular physiology. A gene regulatory network is a dynamic system. Although dynamic models of gene regulatory networks can reveal important information on how a biological process changes from one state to another, or how to respond to environmental stimuli, they require extensive quantitative information, which is currently not generally available in practice. The development of dynamic models of gene regulatory networks is severely compromised by a lack of experimental techniques to measure the dynamic quantities of gene regulatory networks. In practice, a large number of steady-state gene expression measurement data (e.g. gene expression profiles in normal and abnormal tissues) are available. Therefore, revealing information of steady states or quasi steady states of gene regulatory networks using gene expression profiles is of great interest. So the first step toward studying complex biological systems is to develop a general mathematical framework for studying steady states of gene regulatory networks. At present due to incomplete information on the structure of gene regulatory networks it is difficult to identify physically connected gene regulatory networks. However, instead of reconstructing physically connected gene regulatory networks, it may be feasible to model quasi-genetic networks which are defined as the networks that describe the most likely functional relations between the genes in the network.

Here we briefly give an overview of the structure and dynamics of gene regulatory networks. At the lowest level of detail, we distinguish the stoichiometric structure of a regulatory network. It is a description of all regulatory interactions that take place in the network (e.g., of activation, inhibition, transcription, translation and binding). It represents the topology of the flow of gene expression through the network. Linear structural equations can be used for constructing a first order approximation model of a gene regulatory network using steady state gene expression measurements [1]. Let $\mathbf{g}$ denote the expression levels of the genes in the network and $\mathbf{f}$ denote the vector of non-linear functions. Rate equations indicating the expression levels of the genes in the network are given in a simplified form as [2–4].

$$d\mathbf{g}/dt = \mathbf{f}(\mathbf{g}, \mathbf{u}) \tag{1}$$

where $\mathbf{u}$ is the set of transcriptional perturbations. For small perturbations when the system reaches a steady state which is equivalent to setting the time derivative of $\mathbf{g}$ to zero, the non linear system can be approximated by a linear set of equations [5]

$$d\mathbf{g}/dt = \mathbf{B}\mathbf{v} \tag{2}$$

where $\mathbf{B}$ is the node-edge incidence matrix and $\mathbf{v}$ is the vector of flows consisting of both internal and exchange flows. The order of the matrix is $m \times n$ with m as the number of genes and n as the total number of regulatory interactions within a gene regulatory network. That is, the total number of edges is n. An element e_{ik} of matrix $\mathbf{B}$ is -1 (+1) if k-th edge (interaction) exits (enters) the node corresponding to gene g_i. Otherwise, $e_{ik} = 0$. Like the stoichiometric matrix in metabolic networks, the node-edge incidence matrix $\mathbf{B}$ plays a similar role in gene regulatory networks. Equation (2) indicates the change

of expression levels of the gene over time. At steady state, equation (2) reduces to

$$\mathbf{Bv} = 0 \tag{3}$$

which indicates the flow balance equations for the network.

The path diagram consists of nodes representing genes and edges representing their regulatory relationship. The directed edge between node i to node j denotes that protein p_i obtained from gene g_i acts as a transcription factor for gene g_j. In other words, the directed edge indicates the amount of flow of $mRNA_i$ towards regulation of gene g_j through protein p_i. The directed edges can represent either activation or inhibition.

Various genetic networks considered

Genetic network reconstructed from yeast cell cycle data

Models have been built to explore the fundamentals of the regulatory pathways for the cell cycle in yeast. Major events of the cell cycle-DNA synthesis, mitosis and cell division are regulated by a complex network of protein interactions that control the activities of cyclin-dependent kinases. The network can be modeled by a set of nonlinear differential equations and its behavior predicted by computer simulation. Recently, significant improvements in the understanding of genome wide dynamics of the cell cycle regulatory pathways from the values of their transcription factors observed through experiments have been made.

The genetic network shown in Fig. S1 [5] was reconstructed involving the genes Cdc28, Clb1, Clb3, Mcm1, Mcm2, Swi4 using their expression profiles. The network reconstructed from the yeast cell cycle data consists of 6 genes, 6 internal flows $v_1, v_2, \ldots, v_6$ and 4 exchange flows v_7, v_8, v_9 and v_{10} from which the node-edge incidence matrix B can be constructed. The six genes in Fig. S1 play an important role in the M/G1 phase of the yeast cell cycle. The target gene Swi4 in the optimal regulatory pathway plays an important role in the cell cycle progression.

Following the method described in Section Method in the the main text we have generated a set of flow vectors with the starting genes as $Cdc28$ and $Clb1$, and the target gene as $Swi4$. Then the objective function y (equation 4) in the main text is minimized, where z is given by $z = c_5v_5 + c_6v_6 + c_{10}v_{10}$ for the genetic network in Fig. S1. Initially λ should be kept small. For each value of λ, we minimize y, and consider that set of c_i-values corresponding to λ as the final solution, for which y becomes minimum. Here we have obtained the 3 optimal regulatory pathways as $p_1 : g_1 \to g_4 \to g_6$, $p_2 : g_1 \to g_5 \to g_6$, $p_3 : g_2 \to g_5 \to g_6$ which are shown by bold black arrows. The extreme regulatory pathways generated by extreme pathway analysis are the same as shown above.

Transcriptional regulatory network of *E. coli*

Genome scale transcriptional regulatory networks(TRNs) have been reconstructed for well studied organisms such as *E. coli* and *S. cerevisiae*. Recent studies of TRNs have concentrated on the topological structure and its connection with gene regulatory pathways. The transcriptional network of *E. coli* is the most complete experimentally characterized network of a single cell. *E. coli* has become a long standing model organism for the detailed study of small-scale regulatory circuits by virtue of the availability of (1) a large and curated set of experimentally determined regulatory interactions; (2) a tested expression compendium; and (3) a reliable platform for the acquisition of additional expression data.

The regulatory network in Fig. S2 is important in oxidative stress response of plant cells [6] and is also an important component in the acid resistance system of *E. coli* [7]. The gene lpd in Fig. S2B codes for lipoamide dehydrogenase which is a component of the pyruvate and 2-oxoglutarate dehydrogenase complexes. Both the pyruvate and 2-oxoglutarate dehydrogenase complexes play key roles in the metabolism of *E. coli*. The gene lpd also functions as glycine cleavage system L protein. The most complex regulatory circuit is the one for the gene slp, which codes for an outer membrane lipoprotein

induced under carbon starvation and stationary phase [8]. It is regulated by 17 regulators as shown in Fig. S2C. These regulators participate in cellular responses to various environmental conditions, such as oxidative stress (soxRS), acid stress (gadW, gadX, evgA, ydeO and yhiE), cold shock (cspE, cspA) and multiple antibiotic resistance (marA). This underlies the importance of this gene in stress response.

In Fig. S2A, the starting gene is *crp* and the target gene is *gadA*. The starting gene in part B, is fnr and the target gene is *lpd*, and in part C the starting gene is *crp* and the target gene is *slp*. The target gene gadA in part A codes for glutamate decarboxylase, an important metabolic enzyme in the gammaaminobutyric acid (GABA) shunt.

For the genetic network in part A of Fig. S2, the expression for z is $z = c_3v_3+c_5v_5+c_7v_7+c_9v_9+c_{10}v_{10}$. The corresponding expressions for part B is $z = c_2v_2 + c_5v_5 + c_7v_7 + c_8v_8 + c_{10}v_{10}$ and for part C is $z = c_{21}v_{21} + c_{22}v_{22} + c_{23}v_{23} + c_{24}v_{24} + c_{25}v_{25}$. For the genetic network in part A in Fig. S2 the optimal pathway obtained is $p_1 : v_1 \rightarrow v_4 \rightarrow v_9$ as shown by bold black arrows. Similarly, the optimal pathways are $p_1 : v_3 \rightarrow v_8$ for part B and $p_1 : v_2 \rightarrow v_{17} \rightarrow v_{18} \rightarrow v_{24}$ for part C as shown by bold black arrows. The extreme regulatory pathways for Fig. S2A and B are the same as derived from our proposed algorithm. We have obtained a different extreme regulatory pathway by the extreme pathway analysis $v_3 \rightarrow v_{11} \rightarrow v_{12} \rightarrow v_{15} \rightarrow v_{16} \rightarrow v_{22}$ for the genetic network in part C of Fig. S2 as shown by white arrows.

This application is particularly challenging as the model bacterium *E.coli* has direct experimental supports. The pyruvate dehydrogenase (PDH) complex of *E. coli* contains three components, pyruvate dehydrogenase (E1p), dehydrolipoate acyltransferase (E2p), and dihydrolipoate dehydrogenase (E3). The *E. coli* PDH complex is composed of 24 units of E1p (AceE [pyruvate dehydrogenase]), 24 units of E2p (AceF [dehydrolipamide acyltranferase]), and 12 units of E3 (LpdA [dihydrolipoamide dehydrogenase]) [9]. The genes (aceE, aceF, and lpdA) encoding these three enzymes form a single operon, together with the pdhR gene, encoding a self-regulator of this operon, in the order pdhR-aceE-aceF-lpdA. PdhR (Fig. S2B) is an important regulator for the steady state maintenance of the central metabolism for energy production in response to changes in external environmental conditions. In order to identify the whole set of target genes under the control of PdhR, a systematic search in vitro was performed for PdhR-binding sequences in the *E. coli* genome using the newly developed genomic systematic evolution of ligands by exponential enrichment (SELEX) method. The two target genes the ndh gene, encoding NADH dehydrogenase II, and the cyoABCDE operon, encoding the cytochrome bo-type oxidase were identified. Under anaerobic conditions, the expression of ndh is repressed by FNR (regulator of fumarate and nitrate reduction), while the expression of cyo ABCDE is repressed by FNR under anaerobic growth conditions. A detailed analysis of the regulation of ndh and cyoABCDE promoters was performed [10]. Results demonstrated that the ndh and cyoABCDE operons are under the direct control of PdhR. Thus it can be concluded that PdhR (the intermediate gene on the optimal regulatory pathway in Fig. S2B) is a master regulator of the genes involved in the main pathway.

Prostate Genetic network

Fig. S3 represents the differentially expressed genetic network for the data set of the tumor prostate tissues consisting of 10 genes and 10 internal flows [3]. In Fig. S3 the starting gene is $SYPL$ and the target gene is $CAVI$. The expression for z is $z = c_7v_7$. Here we have obtained 1 optimal pathway as $p_1 : v_4 \rightarrow v_7$ as shown by bold black arrows. Comparing with the extreme pathway analysis, the extreme regulatory pathway obtained is the same as that derived by our method. Identification of differentially regulated genetic networks may help us to discover the cause of diseases.

Yeast cell cycle genetic network

Fig. S4 represents the connected genetic network with 10 genes: cell cycle gene Swi4; a glycosyltransferase gene, ALG2, involved in the dolichol pathway and regulated at two critical control points in the G1 phase

of the cell cycle (G0/G1 and START [11]); an essential gene of *Saccharomyces cerevisiae* affecting pre-tRNA processing PTA1 ([12]); a pseudouridine synthetase gene, PUS1, which catalyzes the formation of pseudouridines in tRNAs ([13]); a serine and threonine catabolism gene, CHA1 ([14]); and five other unknown genes for yeast cell cycle gene expression data. In Fig. S4 the starting gene is $ALG2$ and the target gene is $YLL030C$. The expression for z is defined as $z = c_8 v_8 + c_9 v_9$. Here we have obtained 1 optimal pathway as $p_1 : v_2 \to v_6 \to v_8$ as shown by bold black arrows. On comparison with the standard extreme pathway analysis we have obtained the same extreme regulatory pathway as derived by our present method.

Multiple-myeloma (MM) tissue genetic network

Fig. S5 represents the connected differentially regulated genetic network with 8 genes for the multiple-myeloma (MM) tissue data set. It was interesting to note that the gene DF and the receptor AX1 as well as the network were differentially expressed [15]. The most differentially regulated genetic network for the MM data set is shown in Fig. S5. Multiple myeloma (also known as myeloma or plasma cell myeloma) is a progressive hematologic (blood) disease. It is a cancer of the plasma cell, an important part of the immune system that produces immunoglobulins (antibodies) to help fight infection and disease. In Fig. S5 the starting gene is $TRDN$ and the target gene is $GYPB$. For the genetic network in Fig. S5 the expression for z is defined as $z = c_6 v_6 + c_7 v_7$. Here we have obtained 1 optimal pathway as $p_1 : v_1 \to v_2 \to v_5 \to v_6$ as shown by bold black arrows. We have obtained the same extreme regulatory pathway as derived by our proposed method on comparing with the standard extreme pathway analysis.

SOS genetic network

Fig. S6 represents a gene regulatory network for a nine-gene subnetwork of the SOS pathway in *E. coli*. There are nine transcripts in the *E. coli* network to include the principal mediators of the SOS response (lexA and recA), four other regulatory genes with known involvement in the SOS response (ssb, recF, dinI and umuDC), and three sigma factor genes (rpoD, rpoH and rpoS) [16]. In Fig. S6 the starting gene is $lexA$ and the target gene is $rpoD$. The expression for z is defined as $z = c_{11} v_{11} + c_{12} v_{12} + c_{13} v_{13}$. The optimal pathway obtained is $p_1 : v_4 \to v_{12}$ as shown by bold black arrows. We have obtained the same extreme regulatory pathway as derived by our present method on comparing with the standard extreme pathway analysis.

Apoptotic Genetic network (Fig. 1 in the paper)

Apoptosis, or programmed cell death, is a highly regulated process that allows a cell to self-degrade in order for the body to eliminate unwanted or dysfunctional cells. During apoptosis, the genome of the cell will fracture, the cell will shrink and part of the cell will disintegrate into smaller apoptotic bodies. Apoptosis is triggered through two main signaling mechanisms: the intrinsic and the extrinsic pathways [17, 18]. The extrinsic pathway is initiated through the stimulation of the transmembrane death receptors, such as the Fas receptors, located on the cell membrane and when the pro-apoptotic ligand binds to pro-apoptotic receptors on the surface of the cell [19]. In contrast, the intrinsic pathway is initiated through the release of signal factors by mitochondria within the cell and by the cellular developmental cues or as a result of severe cell stress (e.g., DNA damage) [18]. Destruction of the cell is ultimately carried out by caspases, intracellular enzymes that, once activated, destroy cellular proteins that are essential for cell survival.

In the extrinsic pathway, signal molecules known as ligands, which are released by other cells, bind to transmembrane death receptors on the target cell to induce apoptosis. For example, the immune system's natural killer cells possess the Fas ligand (FasL) on their surface [20]. The binding of the FasL to Fas receptors (a death receptor) on the target cell will trigger multiple receptors to aggregate together

on the surface of the target cell. The aggregation of these receptors recruits an adaptor protein known as Fas-associated death domain protein (FADD) on the cytoplasmic side of the receptors. FADD, in turn, recruits caspase-8, an initiator protein, to form the death-inducing signal complex (DISC). DISC in turn activates downstream effecters CASP7 and DFF45, resulting in DNA fragmentation. Through the recruitment of caspase-8 to DISC, caspase-8 will be activated and it is now able to directly activate caspase-3, an effector protein, to initiate degradation of the cell. Active caspase-8 can also cleave BID protein to tBID, which acts as a signal on the membrane of mitochondria to facilitate the release of cytochrome c in the intrinsic pathway.

The intrinsic pathway is triggered by cellular stress, specifically mitochondrial stress caused by factors such as DNA damage and heat shock. Upon receiving the stress signal, the proapoptotic proteins in the cytoplasm, BAX and BID, bind to the outer membrane of the mitochondria to signal the release of the internal content. However, the signal of BAX and BID is not enough to trigger a full release. BAK, another proapoptotic protein that resides within the mitochondria, is also needed to fully promote the release of cytochrome c and the intramembrane content from the mitochondria [21]. Following the release, cytochrome c forms a complex in the cytoplasm with adenosine triphosphate (ATP), an energy molecule, and Apaf-1, an enzyme. Following its formation, the complex will activate caspase-9, an initiator protein. In return, the activated caspase-9 works together with the complex of cytochrome c, ATP and Apaf-1 to form an apoptosome, which in turn activates caspase-3, the effector protein that initiates degradation. The gene CASP3 will again trigger DNA fragmentation factor DFF45 and lead to DNA fragmentation.

These regulatory flows play an important role in apoptosis. The flows from the gene Fas to the gene FADD, and from the gene TRADD to the gene FADD involve responses to DISC [22]. The genes CASP3, FADD, CASP8 and CASP10 are essential in apoptosis. The gene CASP3 is a major effecter gene and carries out the majority of substrate proteolysis during apoptosis [22]. FADD, CASP8 and CASP10 participate in DISC formation and play important roles in apoptosis initiation [23].

Th regulatory network (Fig. 3 in the paper)

Th1 cells are producers of IFN-γ [24], which acts on its target cells by binding to a cell-membrane receptor to start a signaling cascade, which involves JAK1 and STAT-1 [25]. In contrast, STAT-1 plays a role in modulating IL-4, being an intermediate in the negative regulation of IFN-γ exerted on IL-4 expression [26]. SOCS-1 is, a key element for the inhibition from the IFN-γ to the IL-4 pathway. Finally, another key molecule is T-bet, which is a transcription factor detected in Th1 but not Th0 or Th2 cells. T-bet expression is upregulated by IFN-γ in a STAT-1-dependent mechanism [27]. Importantly, T-bet is an inhibitor of GATA-3 [28], an activator of IFN-γ and activator of Tbet itself [29, 30].

References

1. Datta S (2001) RRelationships in Gene Expressions: A Partial Least Squares Approach. Gene Expr 9: 249-255.

2. Gardner TS, di Bernardo D, Lorenz D, Collins JJ (2003) Inferring Genetic Networks and Identifying Compound Mode of Action via Expression Profiling. Science 301: 102-105.

3. Xiong MM, Li J, Fang XZ (2004) Identification of genetic networks. Genetics 166: 1037-1052.

4. de Jong H (2002) Modeling and Simulation of Genetic Regulatory Systems: A Literature Review. Journal of Computational Biology 9(1): 67-103.

5. Xiong M, Zhao J, Xiong H (2004) Network-based regulatory pathways analysis. Bioinformatics 20(13): 2056- 2066.

6. Bouche N, Fromm H (2004) Gaba in plants: just a metabolite? TRENDS in Plant Science 9(3): 110-115.

7. Masuda N, Church GM (2003) Regulatory network of acid resistance genes in *Escherichia coli.* Molecular Microbiology 48(3): 699-712.

8. Rudd K (2000) Ecogene: a genome sequence database for *Escherichia coli.* k-12. Nucleic Acids Res 28(1): 60-64.

9. Faith JJ, Hayete B, Thaden JT, Mogno I, Wierzbowski J, et al. (2007) Large-scale mapping and validation of Escherichia coli transcriptional regulation from a compendium of expression profiles. Plos Biology 5(1): e8. doi:10.1371/journal.pbio.0050008.

10. Ogasawara H, Ishida Y, Yamada K, Yamamoto K, Ishihama A (2007) Pdhr (pyruvate dehydrogenase complex regulator) controls the respiratory electron transport system in *Escherichia coli.* Journal of Bacteriology 189(15): 5534-5541.

11. Lennon K, Pretel R, Kesselheim J, Heesen S, Kukuru-zinska M (1995) Proliferation-dependent differential regulation of the dolichol pathway genes in Saccharomyces cerevisiae. Glycobiology 5(6): 633-642.

12. O'Connor JP, Peebles CL (1992) PTA1, an essential gene of Saccharomyces cerevisiae affecting pre-tRNA processing. Mol Cell Biol 12(9): 3843-3856.

13. Arluison V, Batelier G, Ries-Kautt M, Grosjean H (1999) RNA:pseudouridine synthetase Pus1 from Saccharomyces cerevisiae: oligomerization property and stoichiometry of the complex with yeast tRNA(Phe). Biochimie 81(7): 751-756.

14. Bornaes C, Peterson JGL, Holmberg S (1992) Serine and Threonine Catabolism in Saccharomyces cerevisiae: The CHA1 Polypeptide Is Homologous With Other Serine and Threonine Dehydratases. Genetics 131: 531-539.

15. Sagawa M, Nakazato T, Uchida H, Ikeda Y, Kizaki M (2008) Cantharidin induces apoptosis of human multiple myeloma cells via inhibition of the JAK/STAT pathway. Cancer Science 99(9): 1820-1826.

16. Yamanaka T, Toyoshiba H, Sone H, Parham FM, Portier CJ (2004) The TAO-Gen Algorithm for Identifying Gene Interaction Networks with Applications to SOS Repair in *E.coli.* Environmental Health Perspectives 112(16): 1614-1621.

17. Ghobrial IM, Witzig TE, Adjei AA (2005) Targeting apoptosis pathways in cancer therapy. CA Cancer J Clin 55: 178-194.

18. Jin Z, El-Deiry WS (2005) Overview of cell death signaling pathways. Cancer Biol Ther 4(2): 139-163.

19. Ashkenazi A (2002) Targeting death and decoy receptors of the tumour-necrosis factor superfamily. Nature Reviews Cancer 2: 420-430.

20. Csipo I, Montel AH, Hobbs JA, Morse PA, Brahmi Z (1998) Effect of Fas+ and Fas- target cells on the ability of NK cells to repeatedly fragment DNA and trigger lysis via the Fas lytic pathway. Apoptosis 3(2): 105-114.

21. Hague A, Paraskeva C (2004) Apoptosis and disease: a matter of cell fate. Nature Cell Death and Differentiation 11: 1366-1372.

22. Shivapurkar N, Reddy J, Chaudhary PM, Gazdar AF (2003) Apoptosis and lung cancer: A review. J Cell Biochem 88: 885-898.

23. Kischkel FC, Lawrence DA, Tinel A, LeBlanc H, Virmani A, et al. (2001) Death receptor recruitment of endogenous caspase-10 and apoptosis initiation in the absence of caspase-8. J Biol Chem 276(49): 46639-46646.

24. Murphy KM, Reiner SL (2002) The lineage decisions on helper T cells. Nat Rev Immunol 2: 933-944.

25. Kerr IM, Costa-Pereira AP, Lillemeier BF, Strobl B (2003) Of JAKs, STATs, blind watchmakers, jeeps and trains. FEBS Lett 546(1): 1-5.

26. Elser B, Lohoff M, Kock S, Giaisi M, Kirchhoff S, et al. (2002) Ifn-γ represses IL-4 expression via IRF-1 and IRF-2. Immunity 17(6): 703-712.

27. Lighvani AA, Frucht DM, Jankovic D, Yamane H, Aliberti J, et al. (2001) T-bet is rapidly induced by interferon-γ in lymphoid and myeloid cells. Proc Natl Acad Sci USA 98(26): 15137-15142.

28. Szabo SJ, Kim ST, Costa GL, Zhang X, Fathman CG, et al. (2000) A novel transcription factor, T-bet, directs Th1 lineage commitment. Cell 100(6): 655-669.

29. Mullen AC, High FA, Hutchins AS, Lee HW, Villarino AV, et al. (2001) Role of T-bet in commitment of TH1 cells before IL-12-dependent selection. Science 292(5523): 1907-1910.

30. Zhang Y, Apilado R, Coleman J, Ben-Sasson S, Tsang S, et al. (2001) Interferon gamma stabilizes the T helper cell type1 phenotype. J Exp Med 194(2): 165-172.
