# Supplementary material for: Gradient Descent Optimization in Gene Regulatory Pathways
Source: PLoS One. 2010 Sep 3;5(9):e12475. doi: 10.1371/journal.pone.0012475 (PMC2933224; doi:10.1371/journal.pone.0012475)
Supplement: Table S1 — Some possible pathways with their c-values and z-values for the system in Fig. 1. (0.01 MB PDF) [file pone.0012475.s002.pdf]

TABLE S1

Some possible pathways with their c-values and z-values for the system in Fig. 1

Serial Number	Some possible paths	Optimal c-values	Average quantity (z) of protein synthesis
1	$v_3 \to v_4 \to v_9$ $\to v_{24} \to v_{27}$	$c_3 = 0.92, c_4 = 0.91, c_9 = 0.35$ $c_{24} = 0.27, c_{27} = 0.96$	11.67
2	$v_3 \to v_4 \to v_9$ $\to v_{23} \to v_{26}$	$c_3 = 0.92, c_4 = 0.91, c_9 = 0.35$ $c_{23} = 0.03, c_{26} = 0.85$	15.44
3	$v_3 \to v_4 \to v_{20}$ $\to v_{21} \to v_{27}$	$c_3 = 0.92, c_4 = 0.91, c_{20} = 0.98$ $c_{21} = 0.15, c_{27} = 0.96$	10.51
4	$v_3 \to v_4 \to v_{10}$ $\to v_{20} \to v_{26}$	$c_3 = 0.92, c_4 = 0.91, c_{10} = 0.88$ $c_{20} = 0.98, c_{26} = 0.85$	55.39
5	$v_5 \to v_6 \to v_8$ $\to v_{10} \to v_{16} \to v_{14}$ $\to v_{15} \to v_{18} \to v_{27}$	$c_5 = 0.89, c_6 = 0.96, c_8 = 0.86$ $c_{10} = 0.88, c_{16} = 0.93, c_{14} = 0.88$ $c_{15} = 0.84, c_{18} = 0.93, c_{27} = 0.96$	52.41
6	$v_5 \to v_6 \to v_8$ $\to v_9 \to v_{24} \to v_{27}$	$c_5 = 0.89, c_6 = 0.96, c_8 = 0.86$ $c_9 = 0.35, c_{24} = 0.27, c_{27} = 0.96$	23.72
7	$v_5 \to v_6 \to v_8$ $\to v_9 \to v_{23} \to v_{26}$	$c_5 = 0.89, c_6 = 0.96, c_8 = 0.86$ $c_9 = 0.35, c_{23} = 0.03, c_{26} = 0.85$	19.27
8	$v_5 \to v_6 \to v_8$ $\to v_{10} \to v_{20} \to v_{26}$	$c_5 = 0.89, c_6 = 0.96, c_8 = 0.86$ $c_{10} = 0.88, c_{20} = 0.98, c_{26} = 0.85$	24.51
9	$v_5 \to v_6 \to v_8$ $\to v_{10} \to v_{21} \to v_{27}$	$c_5 = 0.89, c_6 = 0.96, c_8 = 0.86$ $c_{10} = 0.88, c_{21} = 0.15, c_{27} = 0.96$	16.55
