# Supplementary material for: Gradient Descent Optimization in Gene Regulatory Pathways
Source: PLoS One. 2010 Sep 3;5(9):e12475. doi: 10.1371/journal.pone.0012475 (PMC2933224; doi:10.1371/journal.pone.0012475)
Supplement: Table S2 — Variation of c-values and z-values with the upper bound on regulatory flows for the optimal path p1: v3→v4→v10→v20→v26 of the system in Fig. 1. (0.01 MB PDF) [file pone.0012475.s003.pdf]

TABLE S2

Variation of c-values and z-values with the upper bound on regulatory flows for the optimal path $p_1 : v_3 \rightarrow v_4 \rightarrow v_{10} \rightarrow v_{20} \rightarrow v_{26}$ of the system in Fig. 1

Serial Number	Upper bound on flow value	Optimal c-values	Average quantity (z) of protein synthesis
1	5000	$c_3 = 0.93, c_4 = 0.83, c_{10} = 0.91, c_{20} = 0.85, c_{26} = 0.88$	5671.44
2	4000	$c_3 = 0.96, c_4 = 0.95, c_{10} = 0.92, c_{20} = 0.81, c_{26} = 0.97$	4535.12
3	3000	$c_3 = 0.97, c_4 = 0.92, c_{10} = 0.91, c_{20} = 0.85, c_{26} = 0.83$	3456.63
4	2000	$c_3 = 0.94, c_4 = 0.95, c_{10} = 0.97, c_{20} = 0.81, c_{26} = 0.86$	2482.41
5	1000	$c_3 = 0.96, c_4 = 0.98, c_{10} = 0.89, c_{20} = 0.82, c_{26} = 0.88$	1250.49
6	50	$c_3 = 0.94, c_4 = 0.96, c_{10} = 0.81, c_{20} = 0.86, c_{26} = 0.84$	78.31
7	40	$c_3 = 0.96, c_4 = 0.98, c_{10} = 0.86, c_{20} = 0.89, c_{26} = 0.81$	65.72
8	30	$c_3 = 0.92, c_4 = 0.87, c_{10} = 0.85, c_{20} = 0.89, c_{26} = 0.86$	42.55
9	20	$c_3 = 0.96, c_4 = 0.85, c_{10} = 0.88, c_{20} = 0.83, c_{26} = 0.89$	25.69
10	10	$c_3 = 0.97, c_4 = 0.89, c_{10} = 0.84, c_{20} = 0.86, c_{26} = 0.87$	14.63
